# KLF9 regulates miR-338-3p/NRCAM axis to block the progression of osteosarcoma cells

**DOI:** 10.7150/jca.63533

**Published:** 2022-03-28

**Authors:** Yunlu Liu, Kuijing Han, Yulin Cao, Yuxiang Hu, Zengwu Shao, Wei Tong, Yanjiu Han, Yong Liu

**Affiliations:** 1 Department of Orthopedics, Union Hospital, Tongji Medical College, Huazhong University of Science and Technology, Wuhan 430022, China.; 2 Department of Orthopedics, Clinical Medical College of Yangzhou University, Northern Jiangsu People's Hospital, Yangzhou, Jiangsu, 225001, China.

**Keywords:** MiR-338-3p, KLF9, NRCAM, osteosarcoma, GEO, TCGA.

## Abstract

**Background:** MiR-338-3p is revealed to serve as a tumor suppressor in several carcinomas. Whereas, the effect of miR-338-3p in the progression of osteosarcoma has not been explored. The aim of this paper was to analyze the functional influences of miR-338-3p on osteosarcoma progression and the potential mechanism.

**Methods:** The expression of genes and miRNAs in osteosarcoma cells was assessed via western blotting or quantitative reverse transcription-polymerase chain reaction (qRT-PCR). Osteosarcoma cellular proliferation was explored by MTT and EdU incorporation assay. Osteosarcoma cellular migratory and invasive capacity was explored by wound-healing and transwell assay. Bioinformatics approaches were adopted to predict target genes. The relationships between miR-338-3p and neuron‑glial‑related cell adhesion (NRCAM), between kruppel-like factor 9 (KLF9) and miR-338-3p were verified by dual-luciferase reporter assay.

**Results:** We found that miR-338-3p was reduced in osteosarcoma and that higher expression of miR-338-3p suppressed proliferative, invasive and migratory ability of osteosarcoma cells. Furthermore, the result showed that overexpression of NRCAM could reduce the anti-tumor role of miR-338-3p in osteosarcoma cells. In addition, we found that overexpression of KLF9 could enhance the expression level of miR-338-3p in osteosarcoma cells.

**Conclusion:** The KLF9/miR-338-3p/NRCAM axis played a significant role in regulating osteosarcoma progression, which may become a promising therapeutic method for osteosarcoma.

## Introduction

Osteosarcoma is a common, highly malignant bone tumor which has high metastatic potential, occurring mainly in children and adolescents 1, 2. Although survival has been significantly elevated by surgical therapy combined with chemotherapy, the 5 year survival rate of osteosarcoma patients with metastasis remains poor 3. Therefore, it is critical to investigate the mechanism of osteosarcoma progression and to explore novel effective therapeutic targets for osteosarcoma patients.

MicroRNAs (miRNAs, miRs) are endogenous small non-coding RNA molecules (19 to 25 nucleotides) that mediate target gene expression through binding to 3' UTR 4. Numerous studies have revealed the roles of miRNAs in various biological activities including proliferation, apoptosis, angiogenesis, invasion as well as migration 5. Meanwhile, growing researches have reported that dysregulation of miR-338-3p was involved in carcinomas 6-8. Besides, a few studies suggested that TF- miRNA axis played a significant role in the progression of cancers. For example, Cai et al. identified a novel TF-miRNA-mRNA co-regulatory network in pulmonary large-cell neuroendocrine carcinoma 9. Yan et al. discovered mitochondria-related core genes and TF-miRNA-hub mrDEGs network in breast cancer 10. We unveiled that the expression of miR-338-3p was obviously reduced in osteosarcoma in accordance to bioinformatics methods. Whereas, no studies have explored the impact of TF-miR-338-3p on the progression of osteosarcoma.

In this study, we verified that the expression level of miR-338-3p in osteosarcoma cell lines and tissues was downregulated and found that miR-338-3p suppressed the development and metastasis of osteosarcoma by targeting NRCAM directly. In addition, we confirmed that the KLF9/ miR-338-3p/NRCAM axis yielded a critical effect on proliferation, invasion and migration in osteosarcoma. Therefore, these results suggested that KLF9/miR-338-3p/NRCAM axis may function as new anti-cancer targets in osteosarcoma.

## Methods and Materials

### Bioinformatics data evaluation

Firstly, the raw osteosarcoma related miRNAs as well as eligible clinical information was achieved from GEO database. The inclusion criteria: 1) Original experimental studies that were performed to select different miRNAs between tumor tissues/cells and normal tissues/adjacent non-tumor tissues/cells in humans; and 2) each dataset had at least 4 osteosarcoma samples and 4 normal samples. Exclusion criteria: 1) Duplicated or overlapping studies/datasets; 2) single sample studies and certain platforms with various datasets; 3) laboratory studies/datasets on the animal level; 4) non‑microarray studies/datasets; and 5) sequence datasets. Subsequently, the obtained raw miRNA data was normalized with the Robust Multi-Array Average (RMA) and Linear Models for Microarray (LIMMA) algorithm 11. Following that, DESeq2 package 12 was made used of to determine the differentially expressed miRNAs (DEMs) linked to osteosarcoma between the osteosarcoma samples/cells and the control group (P<0.01 and logFC>4 were set as threshold). Afterwards, osteosarcoma DEMs were assessed in accordance to the overlapped analysis of osteosarcoma miRNAs in GSE65071 and GSE28423 sets from GEO database. After this, the identified miRNAs were analyzed on the basis of volcano map employing ggplot package.

### Analysis of miR-338-3p target genes

The four websites software including miRWalk2.0 13, TargetScan6.2 14, miRanda 15 and RNA22 16 were adopted to investigate the possible target genes of miR-338-3p. Afterwards, the differentially expressed genes (DEGs) were assessed according to the overlapped analysis of the predicted target genes, TCGA database (TARGET-OS) and GSE12865 set. The DEGs between the osteosarcoma tissues as well as the non-osteosarcoma tissues were processed across the above selected approach 11. After that, the selected DEGs were analyzed by using volcano map via ggplot package. Finally, PCR assays as well as western blot assay were carried out to validate the result.

### Cell culture and transfection

Human osteosarcoma cell lines (MNNG/HOS, MG-63) and a normal osteoblast cell line (hFOB1.19) were purchased from the Cell Bank of Type Culture Collection of Chinese Academy of Sciences (Shanghai, China). The osteoblast cells hFOB1.19 was cultured in Dulbecco's modified Eagle's medium/ham's F‐12 (DMEM/F‐12; Gibco, Grand Island, NY) containing 10% fetal bovine serum (FBS; Gibco) and 0.3 mg/ml G418. The MNNG/HOS and MG-63 cell lines were grown in Eagle's Minimum Essential Medium (MEM; Gibco) supplemented with 10% FBS, 100‐units/ml penicillin, and 100 mg/ml streptomycin (Gibco). The cells were incubated at 37°C in 5% CO_2_ incubator.

The pcDNA (Vector) as well as pcDNA-NRCAM-1 overexpression (NRCAM-OE) plasmids were provided by GenePharma (Shanghai, China). The pcDNA (Vector) and pcDNA-KLF9 overexpression (KLF9-OE) plasmids were obtained from GenePharma (Shanghai, China). The miR-338-3p mimic, miR-338-3p inhibitor and the mimics negative control (miR-NC) were purchased from GenePharma (Shanghai, China). All transfections were executed by applying Lipofectamine 3000 reagent (Invitrogen, USA) in accordance to the manufacturer's protocols. Then cells were cultured for 48 hours following by collection for the validation of transfection efficiency via quantitative reverse transcription-polymerase chain reaction (qRT-PCR) analysis.

### qRT-PCR

Total RNA from the cells was extracted with the TRIzol reagent (Invitrogen, Carls-bad, CA, USA) and then reversely transcribed into complementary DNA (cDNA) according to the instructions. qRT-PCR was conducted according to SYBR Green Master Mix (Applied Biosyste-ms, Foster City, CA, USA). In addition, primer sequences were presented in **Table 1**. The U6 and GAPDH were applied as internal controls for miRNAs and mRNA, respectively. All PCR assays were carried out in triplicate and relative changes of gene expression levels were measured using 2^-ΔΔCT^ method.

### Western blot

Total protein was extracted from cells with Radioimmunoprecipitation assay (RIPA) Lysis Buffer (Beyotime, Shanghai, China). The concentration of extracted protein was measured applying bicinchoninic acid (BCA) kit (Beyotime, Shanghai, China) 17, 18. After that, the cell lysate was separated using 10% SDS‐PAGE and transferred to polyvinylidene difluoride (PVDF) membranes. Subsequently, the membranes were blocked employing skimmed milk for 1 h followed by incubation of primary antibodies (GAPDH, 1:5000, ab8245, Abcam; NRCAM, 1:10000, ab191418, Abcam; KLF9, 1:2000, ab227920, Abcam) overnight at 4°C. Then, the membranes were incubated with corresponding secondary antibody and detected by chemiluminescence.

### Luciferase reporter assay

MiRWalk2.0 software was employed to predict the possible targeting linkage between miR-338-3p and NRCAM, which was validated by luciferase reporter assay. The transfection of MNNG/HOS, MG-63 cells were performed with PMIR-REPORT luciferase vector with wild-type (WT)- NRCAM-3'UTR or mutant (MUT)- NRCAM-3'UTR, miR-338-3p mimic or miR-338-3p inhibitor (miR-338-3p-KD) or miR-NC. After culture for 48h, luciferase activities were measured in accordance to the Dual-Luciferase Reporter Assay System according to manufacturer's protocols. Each transfection was carried out in 3 independent biological replicates.

### Transwell assay

The effect of miR-338-3p on osteosarcoma cells' invasive and migratory capacity was detected by transwell assay. 1×10^5^ cells were added into the top chamber with serum-free medium. The lower chamber was filled with 20% FBS. After that, cells were incubated for 48 h, the non-invasive cells were removed with a cotton swab. Invasive cells were fixed via 90% of the formaldehyde and then stained through 0.5% crystal violet. Finally, stained cells were counted under microscope (Olympus, Tokyo, Japan).

### Wound healing assay

The transfected cells were seeded in 6-well plates at a high density and incubated to form cell monolayers. The monolayers were scraped in three parallel lines with a 200 μL sterile plastic tip and washed with PBS to remove detached cells. The cells were then cultured in 1% FBS complete medium. To visualize wound healing, images were taken at 0 h and 48 h post-wounding. The percentage of wound closure (Original width-Width after cell migration/Original width) was calculated.

### Cell proliferation assay

3‐(4, 5‐dimethylthiazol‐2‐yl)‐2, 5‐diphenyltetrazolium bromide (MTT) assay was applied to evaluate the cell proliferation ability following the manufacturer's instruction. In brief, transfected cells were added into 96-well plates (2×10^3^ cells/well) and incubated overnight at 37 °C with 5% CO_2_ and incubated for at 1st day, 2nd day, 3rd day, 4th day and 5th day, respectively. At each indicated time point, 20 µl MTT (5 mg/ml) was added into each well and incubated for 4 h. After that, 100 ul DMSO (per well) was added to dissolve the formosan. Then, the ODs were measured with a microplate reader at 490 nm.

The effect of miR-338-3p on osteosarcoma cell proliferation was also measured by 5-ethynyl-2'-deoxyuridine (EdU; Ribobio, China)) incorporation assay as recommended by the manufacturer. The nuclei were visualized by a fluorescence microscope, and the quantitative data were expressed as the percentage of EdU-positive nuclei relative to total number of nuclei counted.

### Chromatin immunoprecipitation (ChIP) assay

ChIP assay was conducted in MNNG/HOS and MG-63 cells via a commercial kit (Millipore, Darmstadt, Germany) according to the manufacturer's instructions. After cross-linking by using 1% formaldehyde for 10 min at 37 °C, the target cells were collected and the mixture was shredded to fragments of 200 bp via sonication. Immunoprecipitation was performed by applying the anti-KLF9 antibody (KLF9, 1:100, ab227920, Abcam) or the control IgG. After purification, qPCR was carried out to assess DNA-binding sites.

### *In vivo* animal study

Male BALB/c nude mice were (5 weeks old) were purchased from Beijing Vital River Laboratory Animal Technology Co., Ltd. and kept under sterile specific pathogen-free conditions. MNNG/HOS cells (5×10^6^ cells) in each group (n=5/group) were respectively subcutaneously injected into the nude mice. Every five days, we detected the tumor longitudinal diameter and latitudinal diameter with caliper. Tumor volume was assessed with the formula: V = 0.5 × D × d^2^ (V, volume; D, longitudinal diameter; d, latitudinal diameter). After 25 days, the mice were sacrificed and tumor tissues were weighted.

The Animal Research Committee of the Academic Medical Center at Huazhong University of Science and Technology approved all aspects of this study (No. S2566).

### Statistical analysis

All the results were expressed as mean ± SD and were evaluated via GraphPad Prism Version 7.0 software. Statistical analyses were conducted with Student's t-test or the analysis of variance (ANOVA). P < 0.05 was considered to be statistically significant.

## Results

### The expression level of miR-338-3p was downregulated in osteosarcoma

**Figure 1:**
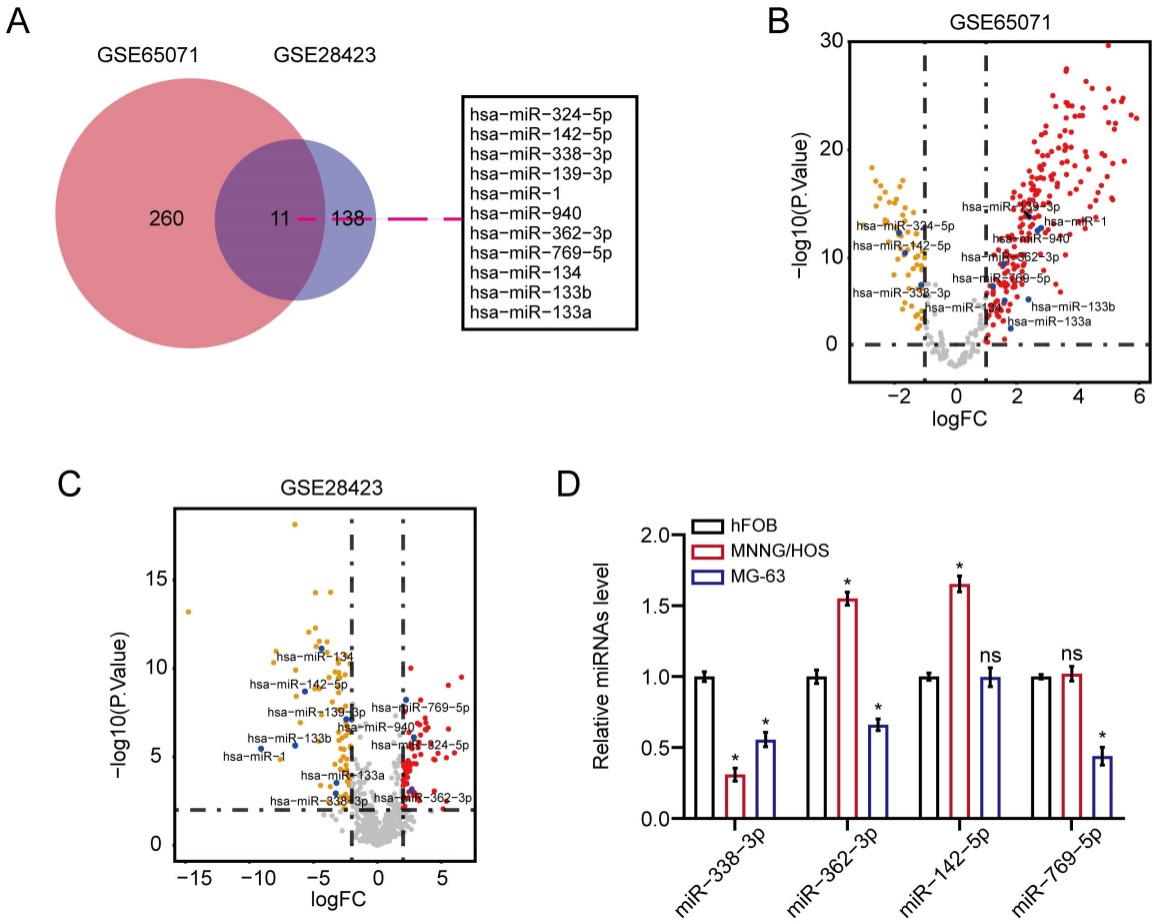
** MiR-338-3p expression was diminished in osteosarcoma.** (A) 11 DEMs were unveiled between osteosarcoma samples/cells and non-osteosarcoma samples/cells. (B, C) the volcano plot of 11 DEMs in GSE65071 and GSE28423 datasets, respectively. (D) qRT-PCR assay was used to assess the expression of miR-338-3p, miR-362-3p, miR-142-5p and miR-769-5p in MNNG/HOS, MG-63 as well as hFOB1.19 cells. Data was presented as mean ± SD from three independent experiments. *, P <0.05; ns, not significant.

We first unearthed osteosarcoma-related DEMs in accordance to the overlapped analysis of osteosarcoma miRNAs from GSE65071 as well as GSE28423 sets of GEO database. As a result, 11 DEMs were unveiled between osteosarcoma samples and non-osteosarcoma samples (Fig. 1A). The result of Figure 1B and 1C showed the volcano plot of 11 DEMs in GSE65071 and GSE28423 datasets, respectively. On the other hand, qRT-PCR assay exhibited that miR-338-3p expression was significantly diminished in MNNG/HOS and MG-63 cells compared with that in hFOB1.19 cells (Fig. 1D).

### Effects of miR-338-3p upregulation on osteosarcoma cells proliferation, invasion and migration

**Figure 2:**
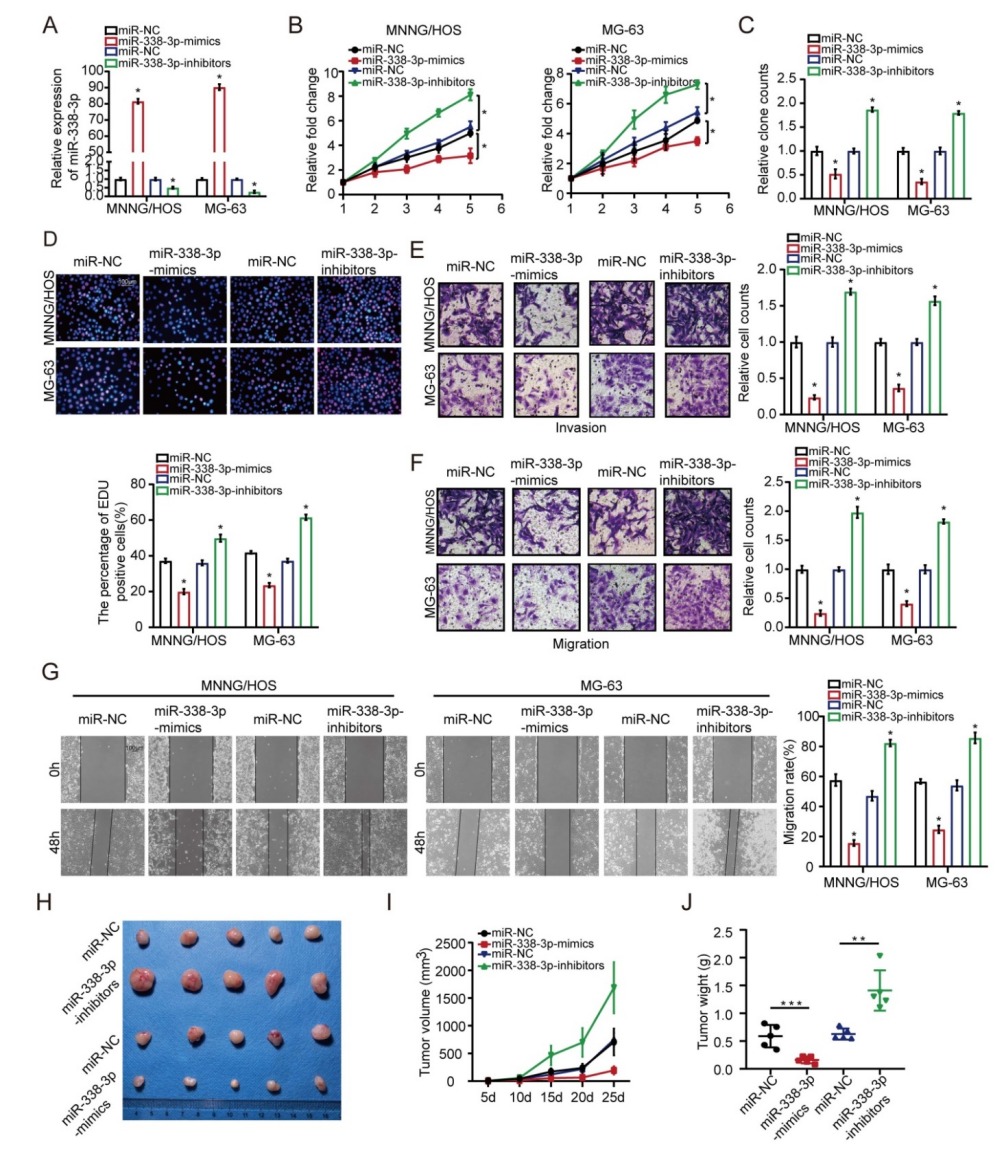
** Effects of miR-338-3p on osteosarcoma cellular proliferation, invasion and migration.** (A) qRT-PCR assay was applied to measure the expression level of miR-338-3p expression with miR-338-3p mimics or miR-338-3p inhibitors in osteosarcoma cells. (B, C) The proliferative ability MNNG/HOS and MG-63 cell was investigated via cell viability and colony formation with miR-338-3p mimics or miR-338-3p inhibitors in osteosarcoma cells. (D) EdU incorporation assays were performed to assess the cell proliferation ability. Scale bars = 100μm. (E, F) Transwell assay was exploited to explore the invasive and migratory ability with miR-338-3p mimics or miR-338-3p inhibitors in osteosarcoma cells. (G) Mobility was evaluated by wound-healing assay. Scale bars = 100μm. (H) Images of tumors after removal from the mice. MNNG/HOS cells were transfected with miR-338-3p mimics or miR-338-3p inhibitors or miR-NC and then injected subcutaneously into nude mice (n=5), respectively. (I) Tumor growth curve. Tumor volumes were measured every five days after injection of tumor cells. (J) Tumor weight when tumors were harvested. Data was presented as mean ± SD from three independent experiments. *, P <0.05.

First of all, MNNG/HOS and MG-63 cells were transfected with miR-338-3p mimics or miR-338-3p inhibitors. Compared to the control group, the expression level of miR-338-3p was significantly increased in the both osteosarcoma cells at 48h post-transfection with miR-338-3p, while miR-338-3p was apparently reduced in the two osteosarcoma cells at 48 h post-transfection with miR-338-3p inhibitors (Fig. 2A). Following that, the effects of miR-338-3p on the migratory and invasive ability of MNNG/HOS and MG-63 cells were assessed through wound-healing and transwell assay. The effects of miR-338-3p on the proliferation ability of MNNG/HOS and MG-63 cells were evaluated by MTT and EdU incorporation assay. The result showed that upregulation of miR-338-3p could significantly block the proliferation, invasion and migration of MNNG/HOS and MG-63 cells. However, the decline of miR-338-3p could significantly promote the proliferation, invasion and migration of MNNG/HOS and MG-63 cells (Fig. 2B-G), indicating that miR-338-3p could inhibit the progression and growth of osteosarcoma. To confirm the role of miR-338-3p in promoting tumor growth *in vivo*, thus, we constructed a subcutaneous xenograft model. The result showed that the tumor size and weight decreased in the miR-338-3p mimics group but increased in the miR-338-3p inhibitors group (Fig. 2H-J).

### NRCAM is the target gene of miR-338-3p in osteosarcoma

**Figure 3:**
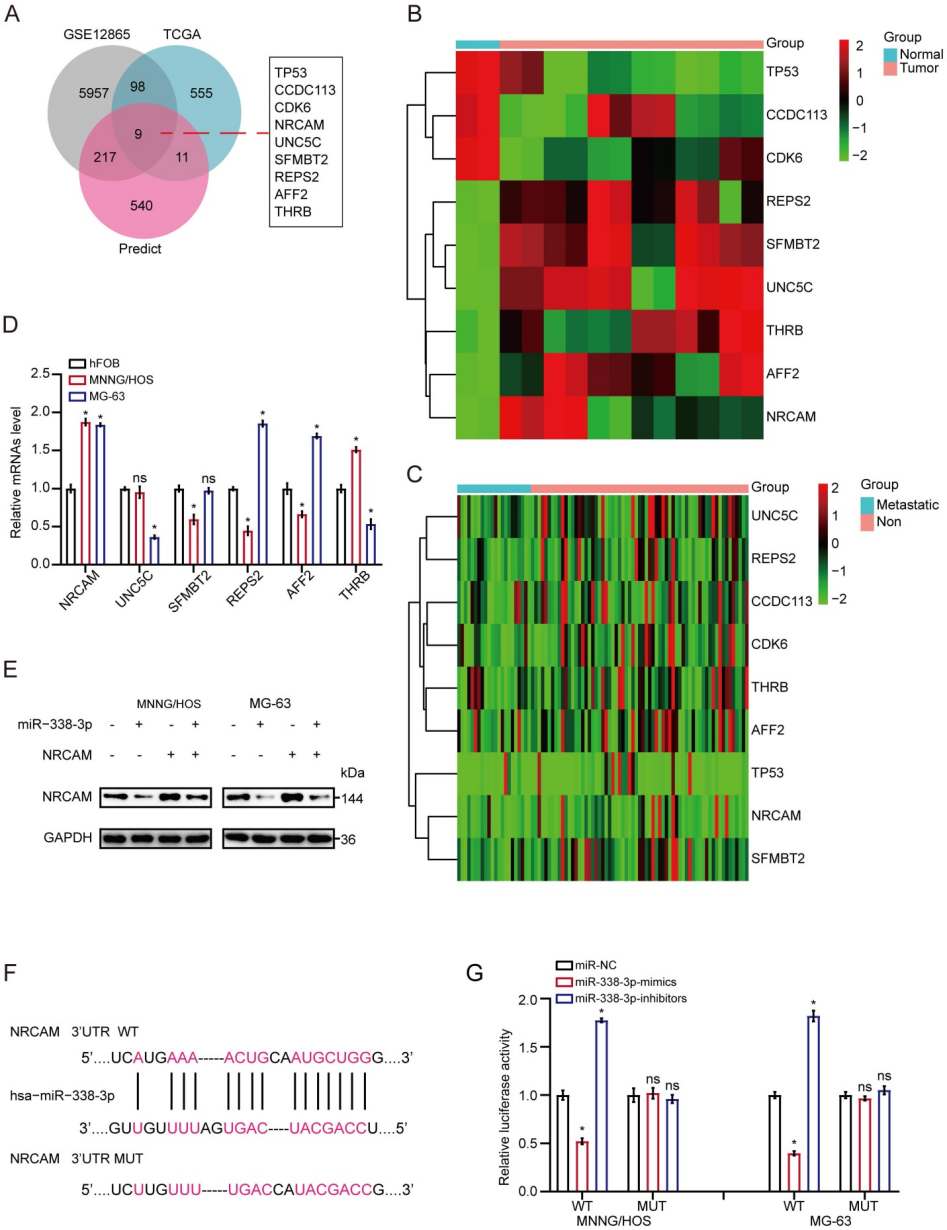
** NRCAM is the target gene of miR-338-3p in osteosarcoma.** (A) Nine common target genes were discovered across the DEGs' overlapping analysis from TCGA and GSE12865 with predicted target genes. (B, C) The nine DEGs' heatmap were showed in GSE12865 set as well as TCGA database, respectively. (D) The six target genes that were obviously raised in both sets were assessed by qRT-PCR assay in MNNG/HOS, MG-63 and hFOB1.19 cells. (E) Western blot assay for NRCAM with miR-338-3p mimics. (F) The descriptive diagram for the binding site of miR-338-3p to NRCAM's 3'-UTR. (G) pMIR-REPORT luciferase vector comprising of NRCAM 3'UTR or a mutated type was co-transfected in osteosarcoma cells with miR-338-3p mimics or inhibitors or miR-NC. Data was presented as mean ± SD from three independent experiments. *, P <0.05; ns, not significant.

**Figure 4:**
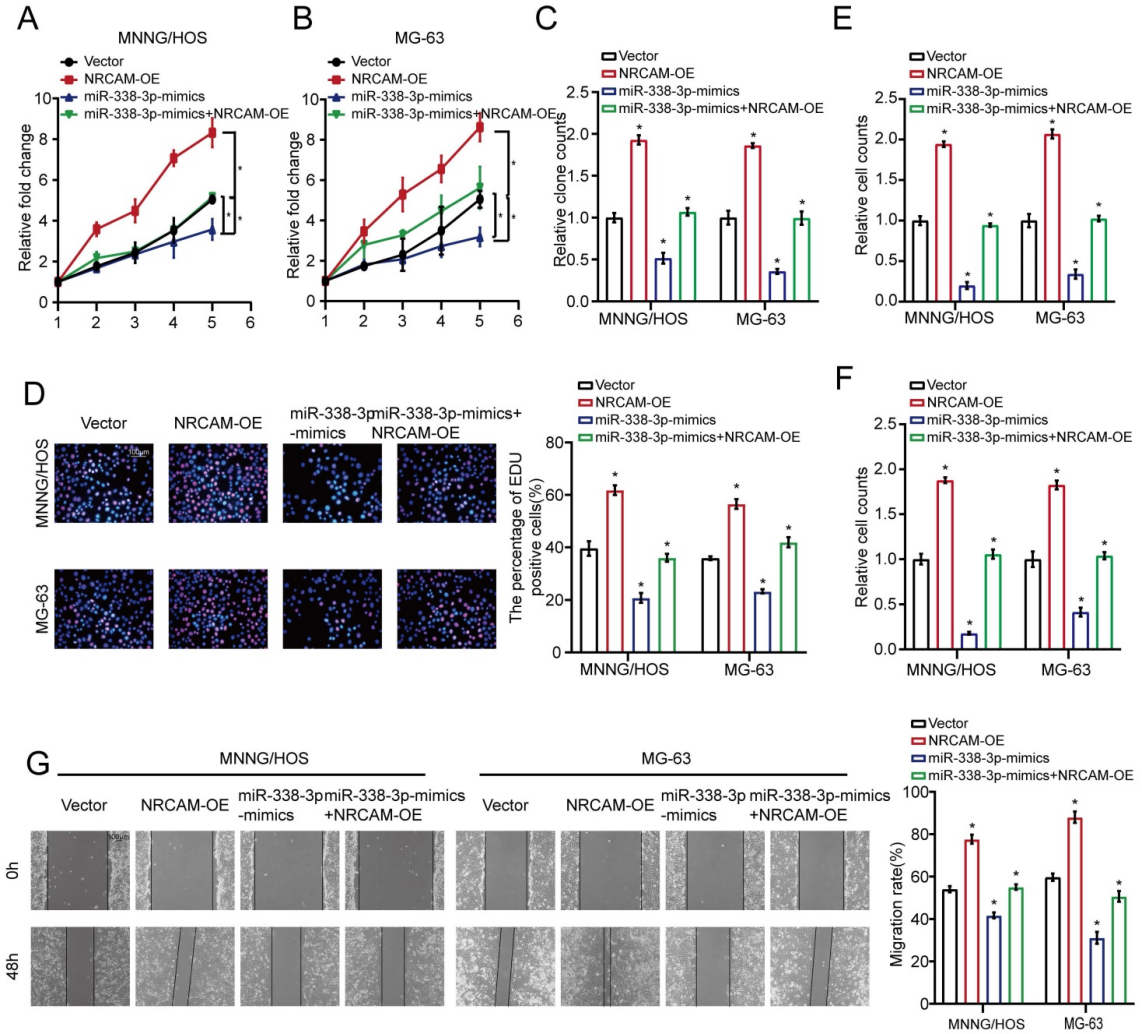
** Overexpression of NRCAM could rescue the enhanced effect of miR-338-3p on osteosarcoma cells.** (A-C) The role of miR-338-3p and NRCAM in the proliferation of osteosarcoma cells was measured across cell viability as well as colony formation. (D) EdU incorporation assays were performed to assess the role of miR-338-3p and NRCAM on cell proliferation ability. Scale bars = 100μm. (E, F) The function of miR-338-3p and NRCAM in the invasion and migration of osteosarcoma cells was analyzed by employing transwell assay. (G) The function of miR-338-3p and NRCAM on osteosarcoma cells mobility were evaluated by wound-healing assay. Scale bars = 100μm. Data was presented as mean ± SD from three independent experiments. *, P <0.05.

Firstly, we implemented the overlapping analysis of osteosarcoma DEGs according to GSE12865, TCGA database as well as predicted target genes. Consequently, nine common target genes were discovered (Fig. 3A). The nine DEGs' heatmaps were observed in GSE12865 set as well as TCGA database, respectively (Fig. 3B and 3C). It is worth noting that, the six out of the above nine target genes were significantly raised in both sets. Interestingly, as for the six target genes, the result of qRT-PCR assay showed that the expression level of NRCAM was raised in MNNG/HOS and MG-63 cells than that in non-osteosarcoma cells (Fig. 3D). Additionally, we analyzed the influence of miR-338-3p on NRCAM level by using western blot analysis. The result indicated that upregulation of miR-338-3p reduced the expression of NRCAM in the both osteosarcoma cells, and the overexpression of NRCAM was also blocked by miR-338-3p mimics (Fig. 3E). miRWalk2.0 was applied to determine potential binding sites between miR-338-3p and NRCAM. The result illustrated that NRCAM mRNA had a 3′-UTR element that was complementary to miR-338-3p (Fig. 3F). Furthermore, the result showed that luciferase activity was reduced in MNNG/HOS and MG-63 cells at the NRCAM-WT-3′-UTR plasmid with miR-338-3p mimics and was raised via miR-338-3p inhibitors. While, MNNG/HOS and MG-63 cells with miR-338-3p over-expression or low-expression had no significant effect in the luciferase intensity of NRCAM-MUT-3'-UTR plasmid (Fig. 3G), illustrating that miR-338-3p targeted on the 3′-UTR of NRCAM. On the other hand, the outcomes also showed that elevated miR-338-3p expression level could suppress the proliferation, invasion and migration of osteosarcoma cells (P<0.05), and this influence could be rescued by NRCAM high-expression (Fig. 4A-G). These collective data showed that miR-338-3p could exert its function in osteosarcoma cells through blocking NRCAM.

### KLF9 target miR-338-3p directly in osteosarcoma cells

**Figure 5:**
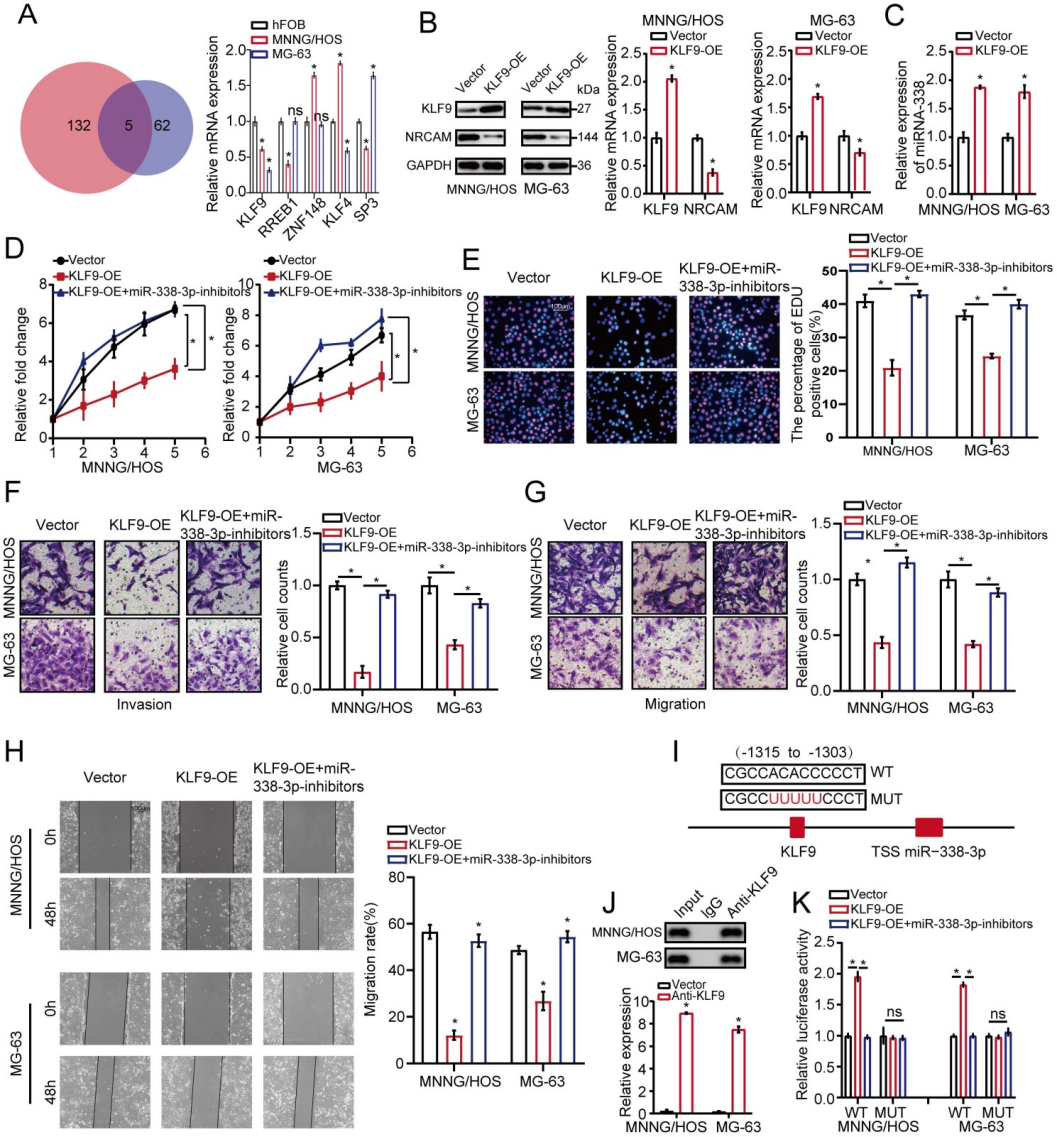
**KLF9 targets miR-338-3p directly in osteosarcoma cells.** (A) qRT-PCR assay was used to assess the expression of the five candidate TFs screened by overlapping DEGs (137) with predicted targets (67). (B) The role of elevated KLF9 expression in NRCAM in osteosarcoma cells was explored by applying western blot assay. (C) The function of raised KLF9 expression on miR-338-3p expression in osteosarcoma cells was investigated by employing qRT-PCR analysis. (D, E) Elevated KLF9 expression could suppress the proliferation of osteosarcoma cells, and the effect may be reversed via diminished miR-338-3p expression. Scale bars = 100μm. (F, G, H) Raised KLF9 expression could significantly inhibit migration and invasion of osteosarcoma cells, which could be rescued by miR-338-3p decline. Scale bars = 100μm. (I) A schematic exhibited the proximal region of the miR-338-3p promoter that was targeted by KLF9. (J) ChIP assay was adopted to validate the binding correlation between KLF9 and the miR-338-3p promoter in osteosarcoma cells. (K) Luciferase activity was increased in the WT miR-338-3p promoter with KLF9 elevation that was clearly rescued via miR-338-3p diminishment. No significant change was unveiled in luciferase activity when KLF9 targeting sites at -1315 to -1303bp were mutated. Data was presented as mean ± SD from three independent experiments. *, P <0.05; ns, not significant.

We then investigated hypothetical miR-338-3p's upstream modulatory molecules. We used JASPAR database as a bioinformatics tool for miR-338-3p's upstream modulatory TFs target prediction, focusing on the putative targets, five candidate TFs were identified by overlapping predicted TFs and DEGs and then were assessed by using qRT-PCR assay. As a result, the expression level of KLF9 was significantly diminished in the both osteosarcoma cells compared with that in hFOB1.19 cells (Fig. 5A). After this, western blot analysis was adopted to assess the effect of KLF9 on NRCAM in the both osteosarcoma cells. The result manifested that NRCAM expression level was obviously diminished with high KLF9 expression in the both osteosarcoma cells (Fig. 5B). We also found that KLF9 overexpression could raise the expression level of miR-338-3p in the two osteosarcoma cells (Fig. 5C). The outcome also showed that high KLF9 expression could inhibit the proliferation, invasion and migration of MNNG/HOS and MG-63 cells, and this effect could be significantly reversed by the decline of miR-338-3p (Fig. 5D-H).

To assess whether KLF9 transcriptionally regulated miR-338-3p, the binding sites of KLF9 in the miR-338-3p promoter region was predicted via JASPAR database. We found that the region at -1315 to -1303bp in upstream of the pre-miR-338-3p promoter region was a target of KLF9 (Fig. 5I and 5J) and constructed two promoter luciferase reporters. The result showed that luciferase activity in the WT miR-338-3p promoter was increased in the two osteosarcoma cells with high KLF9 expression, which was significantly rescued by miR-338-3p inhibitor. Whereas, no alteration was found with mutation of KLF9 bind to site at -1315 to -1303bp in upstream of the pre-miR-338-3p promoter region (Fig. 5K). Taken together, these outcomes indicated that KLF9 could directly regulate the expression and activity of miR-338-3p in osteosarcoma cells.

## Discussion

Numerous studies have emphasized the functions of miRNAs in various biological courses including proliferation, apoptosis invasion as well as migration 5. Several studies have discovered that plenty of miRNAs were abnormally expressed in osteosarcoma which played a critical role in malignant progression 19-21. In this study, we revealed that miR-338-3p was clearly downregulated in the osteosarcoma tissues. While, the effect of miR-338-3p on the mechanism of osteosarcoma remains to be fully explored.

In the present study, we observed the downregulation of miR-338-3p in osteosarcoma and its inhibitory impacts on the proliferation, invasion and migration of osteosarcoma. Several studies have revealed the involvement of miR-338-3p in other kinds of carcinomas. For example, Wang et al. indicated that miR-338-3p targeted RAB23 and suppressed tumorigenicity of prostate cancer cells 22. Zou et al. suggested that miR-338-3p could suppress colorectal cancer proliferation and progression via inhibiting MACC1 23. Zhang et al. demonstrated that miR-338-3p suppressed ovarian cancer cells growth and metastasis 24, which were consistent with our results. Afterwards, we determined that NRCAM was a direct target gene of miR-338-3p in osteosarcoma cells in accordance to bioinformatics methods and a dual luciferase assay. Furthermore, we also found that NRCAM had an enhanced effect on the proliferation, invasion and migration of osteosarcoma cells. A lot of studies revealed that NRCAM was an oncogene. For example, Ling et al. demonstrated that the expression of NRCAM was markedly upregulated in prostate cancer cells in comparison to benign prostate epithelium 25.

Conacci-Sorrell et al. indicated that induction of NRCAM transcription by beta-catenin or plakoglobin played a role in melanoma as well as colon cancer tumorigenesis 26, which were consistent with the outcome of this study. Collectively, the above results demonstrated that overexpression of miR-338-3p might exert its influence on osteosarcoma cells by inhibiting NRCAM.

Plenty of studies demonstrated the involvement of TFs in several cancers. For example, Malik et al. suggested that the TF CBFB suppressed breast cancer via orchestrating translation and transcription 27. Liu et al. revealed TF c-Maf a checkpoint that programed macrophages in lung cancer 28. Jiang et al. indicated that TF NFAT5 contributed to pancreatic cancer progression via transcription of PGK1 29. In our study, we identified TF KLF9 as a significant upstream modulatory factor of miR-338-3p by combined analysis. Subsequently, we analyzed the effect of KLF9 on miR-338-3p and NRCAM expression by using qRT-PCR or western blot assay. In addition, we also verified that high KLF9 expression could inhibit osteosarcoma cells' proliferation, invasion and migration. On the other hand, a few studies reported that KLF9 was tightly involved in cancer. For example, Li et al. revealed that KLF9 could suppress gastric cancer cell invasion and metastasis through transcriptional inhibition of MMP28 30. Fang et al. demonstrated that miR-20a-5p accelerated the proliferation and invasion of non-small cell lung cancer via targeting and downregulating KLF9 31, which were consistent with the outcome of this study. Taken together, we identified that miR-338-3p expression was downregulated in osteosarcoma and that KLF9-miR-338-3p axis could inhibit the proliferation, migration as well as invasion of osteosarcoma cells through targeting NRCAM, which may aid to find a novel therapeutic target for osteosarcoma patients.

Several limitations need to be elaborated in this paper. First of all, the mechanism of KLF9 upregulation remains to be investigated. Additionally, some other genes may act as the downstream target genes of miR-338-3p, which should be studied in the future. In addition, the downstream pathways of NRCAM need to be explored in the future.

## Figures and Tables

**Table 1:** All special primers were enrolled in this study.

Primer	Sequence
miR-338-3p	Forward: 5'-GGGGTACCGAATCTTCCCAGTAGGCG-3'
	Reverse: 5'-TTGCGGCCGCAA AGGAGA AGGGCCAAAC-3'
U6	Forward: 5'-ATTGGAACGATACAGAGAAGATT-3'
	Reverse: 5'-GGAACGCTTCACGAATTTG-3'
GAPDH	Forward: 5'-CAGCCTCAAGATCAGCA-3'
	Reverse: 5'-TGTGGTCAT GAGTCCTTCCA-3'
miR-362-3p	Forward: 5'-GCCGAAACACACCTATTCAAG-3'
	Reverse: 5'-TATGGTTTTGACGACTGTGTGAT-3'
miR-142-5p	Forward: 5'-AACTCCAGCTGGTCCTTAG-3'
	Reverse: 5'-TCTTGAACCCTCATCCTGT-3'
miR-769-5p	Forward: 5'-GGCTGAGACCTCTGGGITC-3'
	Reverse: 5'-CAGTGCGTGTCGTGGAGT-3'
NRCAM	Forward: 5'-GAGCGAAGGGAAAGCTGAGA -3'
	Reverse: 5'-ACAATGGTGATCTGGATGGGC-3'
UNC5C	Forward: 5'- TTACTGGTGCCAGTGTGTGG -3'
	Reverse: 5'-CCAAGGGTTCCTGCTCGAAT-3'
SFMBT2	Forward: 5'-GCGTCGGTGACTAAGCAATC-3'
	Reverse: 5'-CCAATCCCACATAGCGAAGG-3'
REPS2	Forward: 5'-CTGAAGACCAGCAGACACCA-3'
	Reverse: 5'-TTTAGGATCTGGCCCTGTTG-3'
AFF2	Forward: 5'-GCACAAAGCTGATGCACTGT-3'
	Reverse: 5'-GTATGGGGACTTTGCTTCCA-3'
THRB	Forward: 5'-GAACAGTCGTCGCCACATC-3'
	Reverse: 5'-GCTCGTCCTTGTCTAAGTAAC-3'
KLF9	Forward: 5'-ACAGTGGCTGTGGGAAAGTC-3'
	Reverse: 5'-TCACAAAGCGTTGGCCAGCG-3'
RREB1	Forward: 5'-GGGCTTATCCCCCAGTCAAA -3'
	Reverse: 5'-TCTCCGCATCCGACTGACT-3'
MAZ	Forward:5'-CTAACGGGATCCATGTTCCCGGTGTTTCCTTGCACGCTGC-3'
	Reverse:5'-CTAACGGAATTCTCACCAGGGTTGGGAGGGA AGTGGC-3'
ZNF148	Forward: 5'-TGATGATGCCATGCAGTTTT-3'
	Reverse: 5'-TCCCTGCTGTTGTTACT TGCT-3'
KLF4	Forward: 5'‐ACCCACACTTGTGATTACGC‐3'
	Reverse: 5'‐CCGTGTGTTTACGGTAGTGC‐3'
SP3	Forward: 5'‐GGTCAAGTCCAGGTTCAGGG‐3′
	Reverse: 5'‐CTGAGAACTGCCCGAGAGTC‐3′

Abbreviations: AFF2, AF4/FMR2 family member 2; KLF4, krueppel-like factor 4; KLF9, krueppel-like factor 9; MAZ, myc-associated zinc-finger protein; NRCAM, neuron‑glial‑related cell adhesion molecule; REPS2, RalBP1-associated Eps domain-containing protein 2; RREB1, ras-responsive element binding protein 1; SFMBT2, Scm-like with four mbt domains 2; SP3, specificity protein 3; THRB, thyroid hormone receptor beta; UNC5C, unc‑5 netrin receptor C; ZNF148, zinc finger protein 148.
